# Consequences of Radiothermal Ageing on the Crystalline Morphology of Additive-Free Silane-Crosslinked Polyethylene

**DOI:** 10.3390/polym14142912

**Published:** 2022-07-18

**Authors:** Sarah Hettal, Sébastien Roland, Xavier Colin

**Affiliations:** Laboratoire Procédés et Ingénierie en Mécanique et Matériaux, Arts et Métiers Institute of Technology, CNRS, CNAM, HESAM University, 151 Boulevard de l’Hôpital, 75013 Paris, France; sarah.hettal@ensam.eu (S.H.); sebastien.roland@ensam.eu (S.R.)

**Keywords:** silane-crosslinked polyethylene, radiothermal oxidation, chain scissions, chemicrystallisation, lamellar thickening, embrittlement

## Abstract

The radiothermal ageing of silane-crosslinked low-density PE (Si-XLPE) films was studied in the air under three different γ dose rates (8.5, 77.8, and 400 Gy·h^−1^) at a low temperature close to ambient (47, 47, and 21 °C, respectively). Changes in crystalline morphology were investigated using a multi-technique approach based on differential scanning calorimetry (DSC), wide- (WAXS) and small-angle X-ray scattering (SAXS), and density measurements. In particular, the changes in four structural variables were accurately monitored during radiothermal ageing: crystallinity ratio ($X_{C}$), crystalline lamellae thickness ($L_{C}$), long period ($L_{p}$), and interlamellar spacing ($L_{a}$). Concerning the changes in $X_{C}$, a perfect agreement was found between DSC and WAXS experiments. Successive sequences of self-nucleation and annealing (SSA) were also performed on aged Si-XLPE samples in the DSC chamber in order to assess the thickness distribution of crystalline lamellae. This method allowed the thermally splitting of the melting domain of Si-XLPE into a series of elementary melting peaks, with each one characterised by a distinct thickness of crystalline lamellae. DSC (used with the SSA method) showed a slight increase in $L_{C}$ during the oxidation of Si-XLPE, while SAXS confirmed a catastrophic decrease in $L_{a}$. The critical value of the interlamellar spacing characterising the ductile/brittle transition of Si-XLPE was found to be of the same order of magnitude as that for linear polyethylene ($L_{\mathrm{aF}}\approx 6$ nm). This structural end-of-life criterion can now be used for predicting the lifetime of Si-XLPE in a nuclear environment.

## 1. Introduction

Crosslinked low-density polyethylene (XLPE) is widely used as an insulating material for electrical cables in nuclear power plants because of its excellent dielectric properties (e.g., its dipolar moment is zero, and its dielectric constant is around 2.3) [1,2], in addition to its low cost, easy processability, chemical resistance to many chemical reagents, lightness, and great flexibility [3]. However, the main weakness of this material is clearly its fairly high sensitivity to oxidation, which causes its embrittlement [4,5,6,7,8] much earlier than the degradation of its insulating properties [9]. In order to delay the onset of oxidation and, thus, increase its lifetime, antioxidants are commonly incorporated into the polymer matrix during melt processing [10,11,12,13]. However, antioxidants can be physically lost through evaporation and chemically consumed by the reactive species involved in the initiation (i.e., hydroperoxides) and propagation stages (i.e., peroxyl radicals) of the oxidation reaction under service conditions [4,14,15,16,17]. When the XLPE matrix is no longer sufficiently protected, the oxidation rate increases suddenly [4,16,17], thus inducing macromolecular and morphological changes which ultimately lead to the catastrophic decay in fracture properties [4,5,6,7,8].

Therefore, the study of XLPE durability is a multidisciplinary issue that requires addressing scientific challenges at distinct structural scales, typically ranging from molecular to macroscopic scales, including macromolecular and morphological scales. In a nuclear power plant, the oxidation of hydrocarbon polymers can be initiated by both the radiolytic decomposition of C–H bonds and the thermal decomposition of hydroperoxides [18,19]. For this reason, this chemical reaction is commonly called “radiothermal oxidation”. According to the literature, the radiothermal oxidation of XLPE leads to a predominance of chain scissions over crosslinking, which progressively destroys the elastically active chains of the macromolecular network [5,6,7,8] and produces short macromolecular fragments that can rapidly migrate towards crystalline lamellae in order to integrate them, i.e., to initiate chemicrystallisation phenomena [6,7,8]. Of course, such migration is greatly favoured in the case of XLPE because its amorphous phase is in a rubbery state at room temperature. Chemicrystallisation results in a thickening of crystalline lamellae and an increase in crystallinity ratio [6,7,8].

All these degradation mechanisms have extensively been studied in the literature over the last three decades and are now fairly well-understood. Kinetic models derived from these mechanisms are under development and have already started to demonstrate their excellent predictive value for the unfilled XLPE matrix [6]. It should be noted that these models have also been successfully generalised to the alumina tri-hydrate (ATH)-filled XLPE matrix by also taking into account the radiolytic decomposition of the covalent bonds at the ATH/XLPE interface [8]. However, structural end-of-life criteria are still to be determined in order to non-empirically predict the lifetime of XLPE-based materials.

According to the sequential chain described above, the embrittlement of the XLPE matrix could be the consequence of the following two processes:

- Chain scissions lead to deep damage in the macromolecular network, which prevents the amorphous phase from continuing to act as a binder between the crystalline lamellae and distribute the mechanical loading over all the crystalline lamellae;
- Chemicrystallisation leads to a confinement of the amorphous phase, i.e., a loss of molecular mobility leading to a transition of its physical state from rubbery to glassy, which ultimately results in a change in its mechanical behaviour from ductile to brittle.

At the macromolecular scale, the critical concentration of chain scissions responsible for the XLPE embrittlement has recently been estimated to $\left(1.3\pm 0.2\right)\times 10^{-1}$ mol·L^−1^ [8]. It should be highlighted that this end-of-life criterion seems to be independent of the ATH content in the XLPE matrix, which opens up interesting developmental prospects for the lifetime prediction of composite materials. In contrast, to our knowledge, no end-of-life criterion has yet been reported in the literature at the morphological scale, i.e., concerning the existence of a critical value of interlamellar spacing, for the XLPE matrix. In other words, what would be the critical value of interlamellar spacing responsible for the embrittlement of XLPE?

However, such structural end-of-life criteria have already been the subject of abundant studies in the literature for linear polymers and, in particular, for linear polyethylene (PE). According to Fayolle et al. [20,21], the embrittlement of PE would occur when its molar mass reaches the critical value of $M_{\mathrm{WF}}=\left(70\pm 30\right)$ kg·mol^−1^, which would correspond to a critical value of interlamellar spacing of $l_{\mathrm{aF}}=6-7$ nm. These two critical quantities would be independent of the initiation mode of the oxidation reaction, i.e., thermal [20,21], photochemical [22], radiochemical [18], initiated by a chemical attack by chlorinated water disinfectants [23], etc. More recently, slightly higher values ($M_{\mathrm{WF}}\approx 84$ kg·mol^−1^ and $l_{\mathrm{aF}}\approx 8.5$ nm) were determined for high-density polyethylene (HDPE) in immersion in a bleach solution regulated at a free chlorine concentration of 5 ppm, a pH=6.5, and a temperature of 70 °C [24].

Although average critical values seem to emerge for PE, the possible role of side branches (to the PE chain) in controlling the changes in crystalline morphology during oxidative degradation remains a topical issue. Indeed, it is well-known that the concentrations of tie-molecules and entanglements are increased by the introduction of side branches along the PE chain [25,26,27]. These branches delay both the disentanglement and pullout of tie-molecules by acting as protrusions [28,29]. The more difficult it is to disentangle the chains in the amorphous phase (due to the presence of side branches), the better the ductility, the crack growth resistance, and the lifetime of PE [30,31,32]. Of course, these effects should be even more pronounced in the case of crosslinked PE.

In the literature, the wide- (WAXS) and small-angle X-ray scattering (SAXS) methods have often been used to accurately monitor the progress of chemicrystallisation during the oxidative degradation of PE [21,22,24,33]. Coupled with uniaxial tensile testing, these techniques have also been used to assess structural end-of-life criteria for PE, such as critical values of the long period and crystalline lamellae thickness, from which the previous critical values of interlamellar spacing were deduced [21,22,24]. A complementary technique, commonly used to analyse the changes in the shape and temperature position of the main melting peak, is differential scanning calorimetry (DSC). However, for a very long time, this latter technique was not used to its full potential. Indeed, most authors used DSC with the sole purpose of showing an increase in the crystallinity ratio and melting point of PE with the progress of oxidation reaction [7,8,9,24,33].

It took until the end of the last century to see the use of DSC with a more sophisticated procedure, giving access to quantitative information on the distribution of crystalline lamellae in PE samples [34,35,36,37]. Performing successive sequences of self-nucleation and annealing (SSA) in the DSC chamber allowed the thermal splitting of the melting domain into a series of elementary melting peaks and, thus, the description of a given crystalline population with greater precision. Indeed, each elementary peak can be characterised by a distinct structural variable, e.g., a distinct lamellar thickness or crystallinity ratio. During the last two decades, the SSA method has been considerably improved. It was successfully applied to a large number of polymers to characterise their lamellar thickness distribution, but also to analyse the impact of several physicochemical parameters on this distribution, such as tacticity, the distribution of comonomers, the length of comonomer sequences, the distribution of side branches, peroxide crosslinking, the miscibility of polymers in blends, the confinement of amorphous phase, etc. [38]. In addition, satisfying agreements were obtained between the results of X-ray scattering and DSC (used with the SSA method) for PE [39,40]. These are the reasons why SSA has now become a popular method to follow the progress of chemicrystallisation during the oxidative degradation of PE [41,42,43,44,45,46,47].

This article aimed to highlight the changes in the crystalline morphology during the radiothermal ageing in the air at a low temperature close to the ambient of silane-crosslinked low-density polyethylene (Si-XLPE), which is being considered for electrical cable insulation applications in nuclear power plants. DSC was used with the SSA method to access quantitative information on the distribution of crystalline lamellae in Si-XLPE and to deduce the average values of crystallinity ratio ($X_{C}$) and lamellar thickness ($L_{C}$), which were compared with the results of X-ray scattering. In parallel, WAXS and SAXS were used to determine the average values of crystallinity ratio ($X_{C}$) and long period ($L_{p}$), and to deduce the average value of interlamellar spacing ($L_{a}$). All these experimental data allowed for an evaluation of the complementarity of DSC, WAXS, and SAXS techniques, but also an accurate description of the progress of chemicrystallisation during the oxidative degradation of Si-XLPE. In addition, micro-indentation was used to assess the consequences of chemicrystallisation on elastic properties. Finally, the comparison between the results obtained via SAXS and uniaxial tensile testing allowed for the proposal of a structural end-of-life criterion to be introduced into the kinetic models [6,8] recently developed for predicting the lifetime of Si-XLPE in a nuclear environment. The comparison between the values of the end-of-life criterion obtained for PE and Si-XLPE also allowed for an evaluation of the impact of crosslinking on chemicrystallisation kinetics.

## 2. Materials and Methods

### 2.1. Materials

Free-additive Si-XLPE films of about 500 µm thick were directly provided by Nexans NRC (Lyon, France). These films were produced through the extrusion of a linear low-density polyethylene grafted with vinyl tri-methoxy silane side groups (Si-g-LDPE). The chemical crosslinking was then performed via immersion in water at 65 °C for 48 h [48].

As soon as they were received, the Si-XLPE films were analysed by several common laboratory techniques to access key physicochemical and mechanical properties characterising their initial state. The values of all these properties are reported in Table 1.

### 2.2. Radiothermal Ageing Conditions

Radiochemical ageing was performed in the Panoza and Roza facilities at UJV Rez, Czech Republic, using a ^60^Co γ-ray source at a low temperature close to ambient. All the exposure conditions are summarised in Table 2. It should be specified that the radiothermal ageing experiments were performed at three distinct dose rates (8.5, 77.8, and 400 Gy·h^−1^) in order to investigate the effect of dose rate on the global oxidation kinetics and its consequences on crystalline morphology. In addition, an atmosphere cooling system was used to avoid the heating of the samples irradiated with the highest dose rate (400 Gy·h^−1^) above 50 °C, which explains why the temperature of exposure was slightly lower in this case (21 °C instead of 47 °C), compared with the two other exposure conditions (8.5 and 77.8 Gy·h^−1^). The withdrawal times and doses at which the samples were removed from the irradiation facilities are given in the two last columns of Table 2.

### 2.3. Experimental Characterisations

#### 2.3.1. Differential Scanning Calorimetry

##### Global Approach

DSC was used to monitor the changes in the crystalline morphology of Si-XLPE during its radiothermal ageing. DSC thermograms were recorded with a TA Instruments Q1000 DSC calorimeter (TA Instruments, Guyancourt, France) previously calibrated with an indium reference. Film samples with a mass of about 10 mg were introduced into a closed standard aluminium pan and were analysed between −50 °C and 250 °C with a heating rate of 10 °C·min^−1^ under a nitrogen flow of 50 mL·min^−1^. The global crystallinity ratio $X_{C}$ was determined as follows:

(1) $$X_{C}=\frac{∆H_{m}}{∆H_{m0}}\times 100$$

where $∆H_{m}$ is the total area of the melting domain observed between 25 and 125 °C on the DSC thermogram, and $∆H_{m0}$ is the melting enthalpy of the PE crystal. According to the literature, $∆H_{m0}=292$ J·g^−1^ [49].

In addition to $X_{C}$, the temperature positions of the different endothermic peaks were also used to assess the thickness distribution of crystalline lamellae by applying the common Gibbs–Thomson’s relationship. This calculation was made after running the SSA procedure.

##### Successive Sequences of Self-Nucleation and Annealing (SSA)

**Figure 1:**
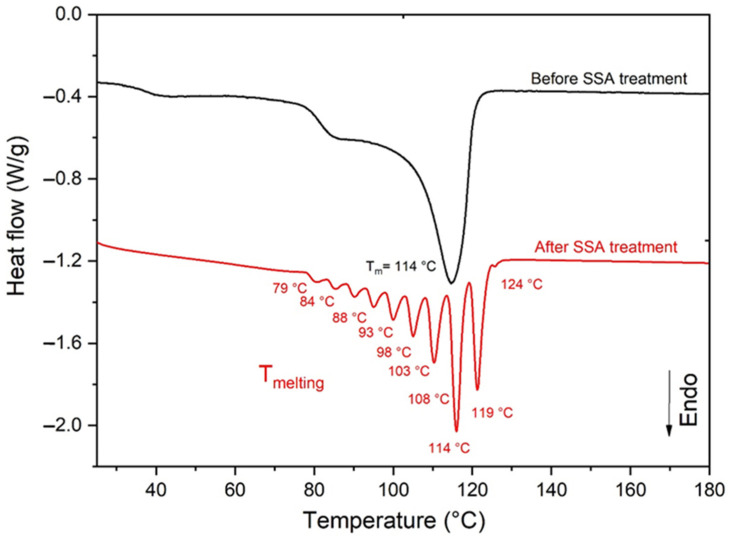
DSC thermograms of the melting peak of unaged Si-XLPE before (in black) and after (in red) running the SSA procedure.

As already explained in the Introduction section, the SSA method allows the thermal splitting of the melting domain of Si-XLPE, typically ranging between 25 and 125 °C on the DSC thermogram, into a series of elementary melting peaks (Figure 1). The set of these peaks represents the population of crystalline lamellae in the Si-XLPE films, each one being characterised by a distinct lamellar thickness $L_{\mathrm{Ci}}$.

Very schematically, the SSA method consists of the following four main steps [34,35,36,37,38]:

(1) First, the sample is heated up to a temperature above its melting point (typically, 250 °C) with a rate of 10 °C.min^−1^ and then held at this temperature for 5 min. This first step aims to erase the thermal history of the sample.
(2) After cooling down at a rate of 10 °C.min^−1^, the sample is heated again up to the self-nucleation temperature $T_{S}$ and then held at this temperature for 5 min. Unmelted crystals undergo annealing while the molten polymer isothermally crystallises. In this study, the temperature $T_{S}$ was determined by using the methodology proposed by Fillon et al. [34]. Of course, a different value of $T_{S}$ was obtained for each ageing state.
(3) Step 2 is repeated nine times by modifying $T_{S}$ by subtracting 5 °C from T_S_ for each cycle, i.e., $T_{S2}=T_{S}-5\;{}^{\circ}C$, $T_{S3}=T_{S2}-5\;{}^{\circ}C$, … etc. until $T_{S10}=T_{S9}-5\;{}^{\circ}C$. Then, the sample is cooled down at a rate of 10 °C·min^−1^ up to room temperature.
(4) Finally, the sample is again heated up to a temperature above its melting point (250 °C) with a rate of 10 °C·min^−1^. This last temperature ramp reveals the structural changes caused by the different annealing treatments performed on the sample, i.e., the splitting of the initial broad melting peak into a series of elementary melting peaks, as presented in Figure 1.

Running the SSA procedure allowed obtaining a fragmented thickness distribution of crystalline lamellae. Within this distribution, each crystalline lamella was characterised by its own melting temperature $T_{\mathrm{mi}}$. Then, the common Gibbs–Thomson’s equation was applied to each melting peak to deduce the corresponding lamellar thickness $L_{\mathrm{Ci}}$:

(2) $$L_{\mathrm{Ci}}=\frac{2\sigma }{\rho_{c}∆H_{m0}}\frac{T_{m0}}{T_{m0}-T_{\mathrm{mi}}}$$

where σ is the crystal surface energy, $T_{m0}$ is the equilibrium melting temperature, and $\rho_{c}$ and $∆H_{m0}$ are the density and the melting enthalpy of the crystalline phase, respectively. The values of these different quantities are available in the literature for PE [49]: $\sigma =7\times 10^{-2}$ J·m^−2^, $T_{m0}=415$ K, $\rho_{c}=1000$ kg·m^−3^ and $∆H_{m0}=292$ J·g^−1^.

Finally, the average lamellar thickness was calculated as follows:

(3) $$L_{C\;}=\frac{∆H_{m1}}{∆H_{m}}L_{C1}+\frac{∆H_{m2}}{∆H_{m}}L_{C2}+\cdots +\frac{∆H_{m10}}{∆H_{m}}{\;L}_{C10}$$

where $∆H_{\mathrm{mi}}$ is the area under each elementary melting peak i, and $∆H_{m}$ is the total area of the melting domain observed between 25 and 125 °C on the DSC thermogram.

#### 2.3.2. X-ray Scattering

##### Wide-Angle X-ray Scattering

**Figure 2:**
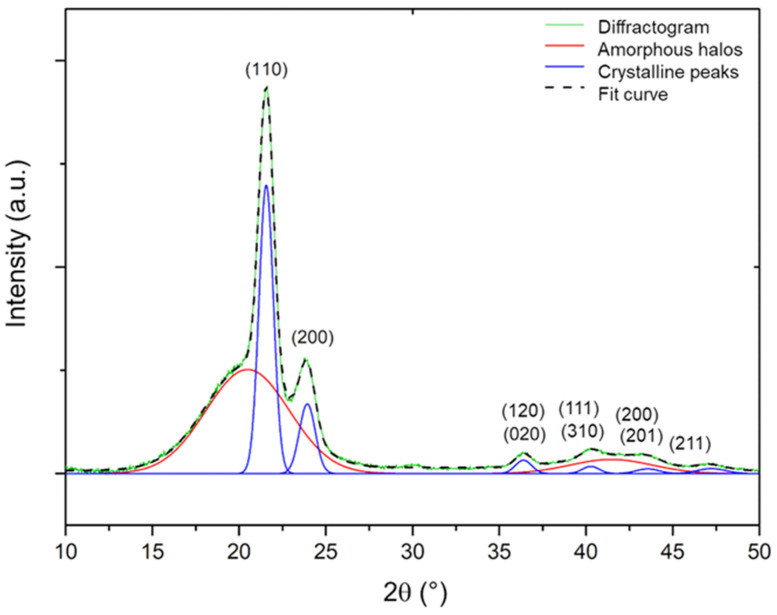
X-ray diffractogram of unaged Si-XLPE (in green) and its several components coming from its deconvolution with the Fityk commercial software: crystalline peaks (in blue) and amorphous halos (in red). The dotted black line corresponds to the sum of the amorphous halos and the crystalline peaks.

WAXS was used to identify and globally quantify the crystalline phase of the Si-XLPE films. The experiments were conducted with a PANalytical X’Pert X-ray diffractometer (PANalytical, Almelo, The Netherlands). The incident beam was composed of monochromatic Co Kα radiation with wavelength λ=1.79 Å. The measurements were conducted with a 2θ angle ranging from 10° to 50°. As an example, Figure 2 reports the raw X-ray diffractogram obtained for the unaged Si-XLPE film.

As shown in Figure 2, all the diffractograms (in green colour) were mathematically deconvolved into two components: crystalline peaks (in blue colour) and amorphous halos (in red colour), using the Fityk commercial software [50]. In fact, these diffractograms exhibited six main crystalline peaks located at angles of 2θ=24°, 27°, 36°, 42°, 43°, and 47° that were respectively assigned to the diffraction planes (110), (200), (120), (111), (201), and (211) of the PE orthorhombic lattice [51]. In addition, they contained two amorphous halos typically ranging between 2θ=15° and 27°, and between 2θ=37° and 45°. 

The global crystallinity ratio $X_{C}$ of the Si-XLPE films was determined as follows:

(4) $$X_{C}=\frac{\sum^{\;}A_{C}}{\sum^{\;}A_{C}+\sum^{\;}A_{a}}$$

where $A_{C}$ and $A_{a}$ are the total areas under the crystalline peaks and the amorphous halos, respectively.

##### Small-Angle X-ray Scattering

SAXS was used to further characterise the polymer crystalline morphology through the determination of the long period $L_{P}$. Indeed, this length is defined as follows:

(5) $$L_{P}=L_{C}+L_{a}$$

where $L_{C}$ corresponds to the average thickness of crystalline lamellae, and $L_{a}$ corresponds to the (amorphous) interlamellar spacing.

The experiments were conducted with several pieces of equipment—namely, an X-ray generator of Genix Xenocs type (Xenocs, Grenoble, France) with a voltage of 50 kV and intensity of 0.7 mA, an anti-scattering slit tube, and a two-dimensional CCD analyser MARresearch 300 2D (MARresearch GmbH, Norderstedt, Germany). The Si-XLPE films were placed between the source and the detector at a distance of 1273.5 mm from the detector, which was determined through calibration with a silver behenate standard.

The intensity of the pattern I was integrated in order to obtain the diffusion curves as a function of the scattering vector q using the Foxtrot commercial software. The intensity was then corrected using the common Lorentz correction as follows:

(6) $$I_{c}=I\left(q\right)\times q^{2}$$

The scattering vector q was determined as follows:

(7) $$q=\frac{4\pi \sin\theta }{2\theta }$$

where 2θ is the scattering angle.

The long period $L_{P}$ was determined at the maximum of the correlation peak as follows:

(8) $$L_{P}=\frac{2\pi }{q_{\max\mathrm{corrected}}}$$

where $q_{\max\mathrm{corrected}}$ is the value of the scattering vector taken at the maximum of the correlation peak. 

The integral of the corrected curve $I_{c}=f\left(q\right)$ is called the invariant Q and is defined as follows:

(9) $$Q=\int^{\;}I\left(q\right)q^{2}d\left(q\right)$$

Semi-crystalline polymers are organised into amorphous and crystalline domains. The invariant Q is proportional to the square of the difference in the electronic densities between the amorphous $\rho_{a}^{e}$ and crystalline phases $\rho_{c}^{e}$.

(10) $$Q={\left(\rho_{c}^{e}-\rho_{a}^{e}\right)}^{2}\times V_{C}\left(1-V_{C}\right)$$

where $V_{C}$ is the volume fraction of crystalline domains and is proportional to the weight fraction of the crystalline phase $X_{C}$ such as:

(11) $$V_{C}=\frac{\rho }{\rho_{c}}{\;X}_{C}$$

where ρ and $\rho_{c}$ are the densities of Si-XLPE and its crystalline phase, respectively. Therefore, density measurements were necessary to access these last parameters.

#### 2.3.3. Density Measurements

The density of the Si-XLPE films was determined through hydrostatic weighing at room temperature (23 °C) using a Mettler Toledo MS104TS microbalance (Mettler Toledo SAS, Viroflay, France). Rectangular film samples were weighed in air and then after immersion in ethanol, and their density was determined by applying the Archimedes principle as follows:

(12) ρ=mAirmAir−mIm ρEth

where $m_{\mathrm{Air}}$ and mIm are the sample weights in air and in ethanol, respectively, and $\rho_{\mathrm{Eth}}$ is the density of ethanol at 23 °C ($\rho_{\mathrm{Eth}}=0.789$ [52]).

The density of the amorphous phase $\rho_{a}$ was deduced as follows:

(13) $$\rho_{a}=\rho \;\times \frac{1-X_{C}}{\frac{1-\rho }{\rho_{C}}\times X_{C}}$$

where $\rho_{C}$ is the density of the crystalline phase ($\rho_{C}=1.014$ [53]).

#### 2.3.4. Micro-Indentation

The consequences of radiothermal oxidation on the elastic properties of the Si-XLPE films were determined via micro-indentation. The films were cut in their thickness direction and embedded in a commercial acrylic KM-V resin which was crosslinked for 12 h under primary vacuum at room temperature. Then, the film cross-sections were polished with a MECAPOL P320 device (PRESI France, Eybens, France) using silicon carbide abrasive papers of decreasing particle size (typically from 80 to 2400 granulometry). Finally, a mirror finish was obtained using diamond pastes of decreasing particle size (typically from 3 to 0.25 μm).

The indentations were performed on the polished cross-sections using an Anton Paar Micro Hardness Indenter (Anton Paar, Les Ulis, France) equipped with a Vickers diamond tip of pyramidal geometry, with a force of 450 mN and a loading and unloading rate of 1000 μm·min^−1^. A pause of 10 s was systematically applied between loading and unloading to eliminate the viscous response of the Si-XLPE matrix. The Indentation 4.37 operating software directly yielded the value of the reduced modulus $E_{\mathrm{red}}$ of the material, which was calculated according to the Oliver–Pharr’s method [54,55,56]:

(14) $$E_{\mathrm{red}}=\frac{\sqrt{\pi }\frac{∆F}{∆h}}{2\beta \sqrt{A_{c}}}$$

where ∆F/∆h is the slope at the origin point of the unloading curve, β is a shape factor depending on the indenter type (β= 1.012 for a Vickers tip), and $A_{c}$ is the contact area between the indenter and the sample, projected perpendicularly to the indenter axis on the sample surface: $A_{c}=a^{2}$, where a is the side length of the projected square. This last quantity is also directly provided by the operating software. It depends both on the penetration depth of the indenter and the indenter geometry.

The local elastic modulus E(j) was deduced from the reduced modulus $E_{\mathrm{red}}$ as follows:

(15) $$E\left(j\right)=\frac{1}{\frac{1-\vartheta^{2}}{E_{\mathrm{red}}}-\frac{1-\vartheta_{\mathrm{ind}}{}^{2}}{E_{\mathrm{ind}}}}$$

where ϑ is the Poisson’s ratio of the unaged Si-XLPE (ϑ= 0.42), and $\vartheta_{\mathrm{ind}}$ and $E_{\mathrm{ind}}$ are the Poisson’s ratio ($\vartheta_{\mathrm{ind}}=$ 0.07) and Young’s modulus of the diamond tip ($E_{\mathrm{ind}}=$ 1141 GPa), respectively.

Profiles of elastic modulus were determined throughout the film thickness with an indentation step of 50 µm. Then, the average elastic modulus of each film E was deduced by averaging the N local values E(j) constituting the micro-indentation profile as follows:

(16) $$E=\frac{1}{N}\overset{N}{\underset{j=1}{\sum }}E\left(j\right)$$

## 3. Results and Discussion

### 3.1. Effect of Radiothermal Ageing on the Microstructure

**Figure 3:**
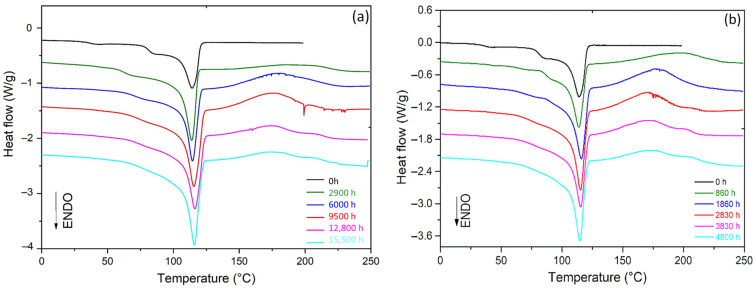

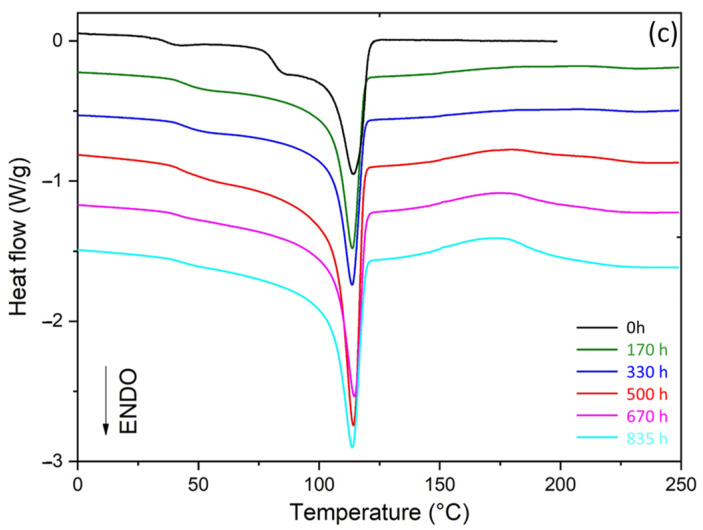
DSC thermograms of Si-XLPE before and after its radiothermal ageing in air under (**a**) 8.5 Gy·h^−1^ at 47 °C, (**b**) 77.8 Gy·h^−1^ at 47 °C, and (**c**) 400 Gy·h^−1^ at 21 °C.

The microstructure of the Si-XLPE films was first characterised before and after radiothermal ageing using DSC. Figure 3 shows the DSC thermograms obtained before and after the radiothermal exposure in the air under 8.5 Gy·h^−1^ at 47 °C, 77.8 Gy·h^−1^ at 47 °C, and 400 Gy·h^−1^ at 21 °C.

The thermogram of the initial sample showed a rather broad melting peak, which typically extended from 25 °C to 125 °C with the presence of two shoulders (at about 40 °C and 80 °C). This result suggested the presence of several crystal populations. It was clearly observed that the amplitudes of these shoulders decreased with the time of exposure, suggesting that radiothermal ageing promotes the formation of a single population of primary lamellae. In addition, the oxidation of Si-XLPE led to the early appearance and rapid growth of an exothermic peak above the melting zone, typically ranging between 130 °C and 230 °C. This new peak corresponded to the thermal decomposition of hydroperoxides under nitrogen in the DSC chamber. In previous papers [6,8], it was used to titrate the hydroperoxides generated during the radiothermal ageing of Si-XLPE and, thus, to access its oxidation kinetics.

**Figure 4:**
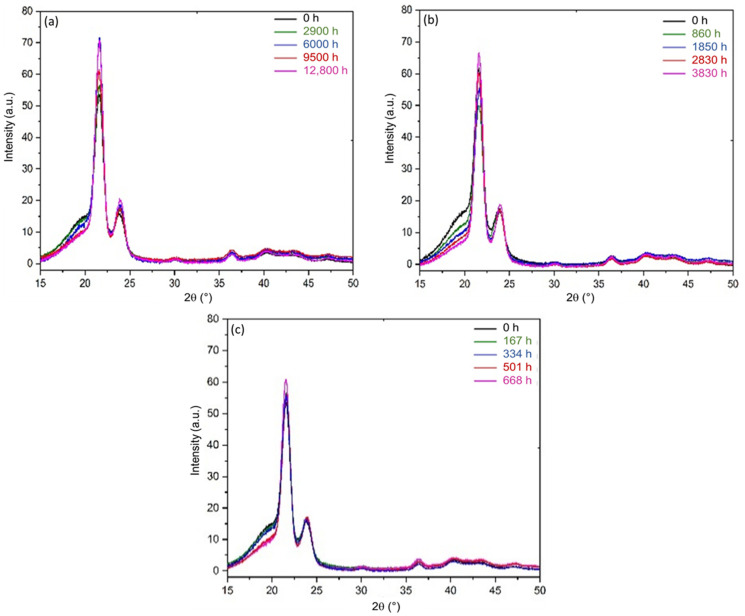
X-ray diffractogram of Si-XLPE before and after its radiothermal ageing in air under (**a**) 8.5 Gy·h^−1^ at 47 °C, (**b**) 77.8 Gy·h^−1^ at 47 °C, and (**c**) 400 Gy·h^−1^ at 21 °C.

WAXS measurements were performed in order to determine the global crystallinity ratio of the Si-XLPE films and to monitor the dimension of the crystalline unit cell during radiothermal ageing. Figure 4 shows the X-ray diffractograms of the Si-XLPE films before and after radiothermal exposure in the air under 8.5 Gy·h^−1^ at 47 °C, 77.8 Gy·h^−1^ at 47 °C, and 400 Gy·h^−1^ at 21 °C. For all dose rates, it is noteworthy that the angular position of the crystalline peaks remained constant. This result showed that the dimension of the unit cell remained unchanged during the radiothermal exposure, which seems to be different from the previous observations made by Badr et al. [57]. Nonetheless, our results suggested that the degradation mechanism occurs only in the amorphous phase, thus leaving the crystalline unit cell unaffected by radiothermal ageing. The density of the crystal could then be considered constant over time of exposure for the three radiothermal ageing conditions under study. In addition, it was observed that the amorphous halo located between 2θ=15° and 25° decreased with the time of exposure, while the crystalline peaks located at 2θ=22° and 24° increased. This result indicated that the crystalline phase grows over the amorphous phase through a crystallisation process.

**Figure 5:**
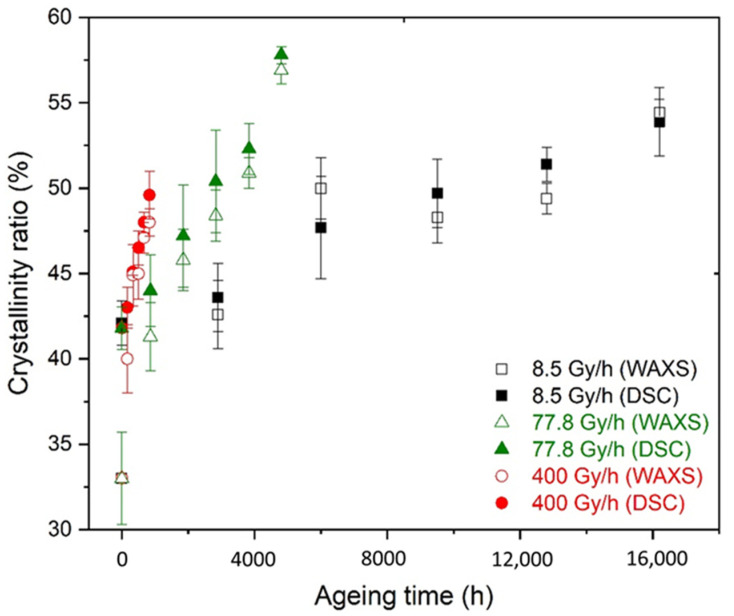
Changes in the crystallinity ratio determined by using DSC and WAXS for Si-XLPE during its radiothermal ageing in air under 8.5 Gy·h^−1^ at 47 °C (in black), 77.8 Gy·h^−1^ at 47 °C (in green), and 400 Gy·h^−1^ at 21 °C (in red).

The changes in the crystallinity ratio during radiothermal ageing were plotted for both DSC and WAXS analyses, and the results are shown in Figure 5. For both techniques, it was observed that the crystallinity ratio increased with the time of exposure. In addition, the results obtained via DSC and WAXS measurements perfectly correlated. The only discrepancy was detected for the initial sample and at the very early stage of ageing. Indeed, the initial crystallinity ratio obtained using DSC was equal to about 42%, whereas a crystallinity ratio of 34% was measured through WAXS. This discrepancy might be due to the initial presence of small crystals in the Si-XLPE films, which disappeared over time of exposure. These small crystals were thermally detected through DSC but were too small (L_C_ = 1.9 nm) to contribute to the diffraction pattern in Bragg’s conditions at room temperature. This assumption was confirmed by calculating the crystalline ratio X_C_ via DSC without integrating the shoulder at around 40 °C. Using this method, a similar value of X_C_ (34%) was measured through DSC and WAXS.

As the time of exposure increased, the population of the small crystals tended to disappear because the values of X_C_ measured through DSC and WAXS became similar. For the dose rates under study, the crystallinity ratio increased over time of exposure, which was probably due to chemicrystallisation phenomena. Indeed, in previous publications [6,8], it was shown that chain scissions predominate largely over crosslinking during the radiothermal ageing of Si-XLPE owing to measurements of gel content and storage modulus at a rubbery plateau. Thus, the elastically active chains of the macromolecular network are progressively destroyed, producing short linear macromolecular fragments. As the amorphous phase of Si-XLPE is in a rubbery state at room temperature, these fragments have high mobility and, thus, can easily migrate towards the surface of crystalline lamellae to induce chemicrystallisation, i.e., lamellar thickening and an increase in crystallinity ratio.

**Figure 6:**
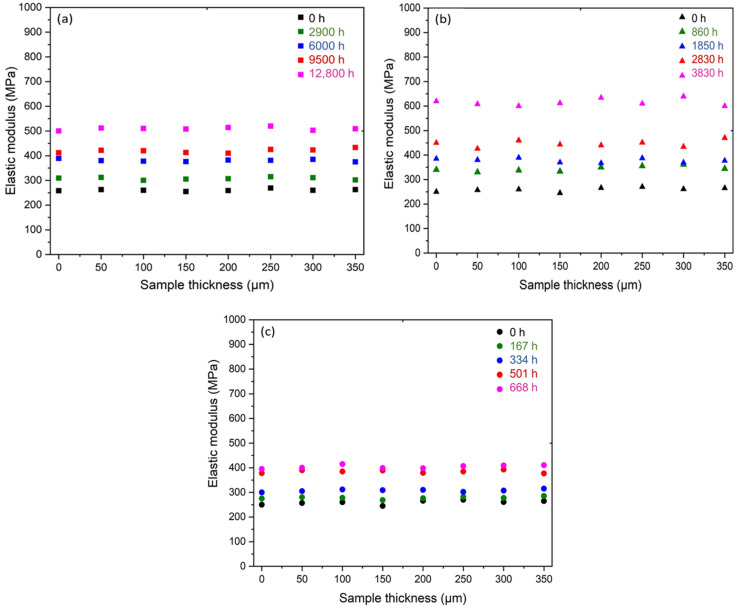
Changes in the profile of the elastic modulus in the sample thickness of Si-XLPE films before and after their radiothermal ageing in air under (**a**) 8.5 Gy·h^−1^ at 47 °C, (**b**) 77.8 Gy·h^−1^ at 47 °C, and (**c**) 400 Gy·h^−1^ at 21 °C.

Micro-indentation measurements were performed in order to determine the consequences of radiothermal ageing on the local elastic properties of the Si-XLPE films throughout their thickness. Figure 6 shows the changes in the profile of the elastic modulus during the radiothermal exposure in the air under 8.5 Gy·h^−1^ at 47 °C, 77.8 Gy·h^−1^ at 47 °C, and 400 Gy·h^−1^ at 21 °C. It is noteworthy that the profile remained flat throughout the thickness for the three radiothermal exposure conditions under study. This is typically the situation where the dose rates are too low to create heterogeneous oxidation in thin Si-XLPE films of about 500 µm thick. In such a case, oxygen has plenty of time to diffuse up to the centre of the sample before being consumed by the oxidation reaction, producing chain scissions and causing chemicrystallisation. In other words, the control of the oxidation kinetics via oxygen diffusion could be neglected.

**Figure 7:**
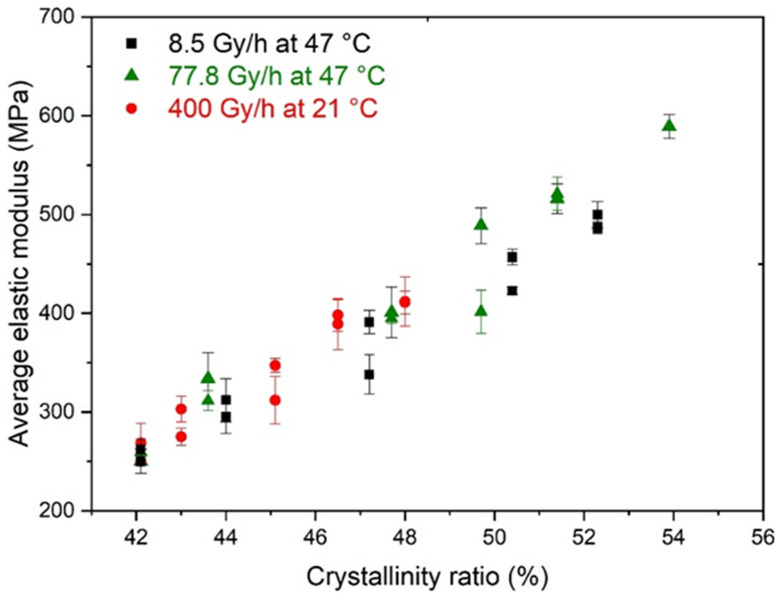
Changes in the average elastic modulus versus crystallinity ratio during the radiothermal ageing of Si-XLPE in air under 8.5 Gy·h^−1^ at 47 °C (in black), 77.8 Gy·h^−1^ at 47 °C (in green), and 400 Gy·h^−1^ at 21 °C (in red).

As the profile of elastic modulus was flat, an average value could easily be calculated. Figure 7 shows the changes in the average elastic modulus E as a function of the crystallinity ratio X_C_. A nice linear correlation was observed between both quantities, which is a classical behaviour for semi-crystalline polymers above their glass transition temperature such as PE [58]. Thus, there is no doubt that the changes in the crystalline morphology of Si-XLPE were mainly responsible for such a correlation. In the following paragraphs, these changes in crystalline morphology are carefully and accurately analysed in order to propose a structural end-of-life criterion for Si-XLPE.

### 3.2. Changes in L_C_, L_P_, and L_a_ during the Radiothermal Ageing

**Figure 8:**
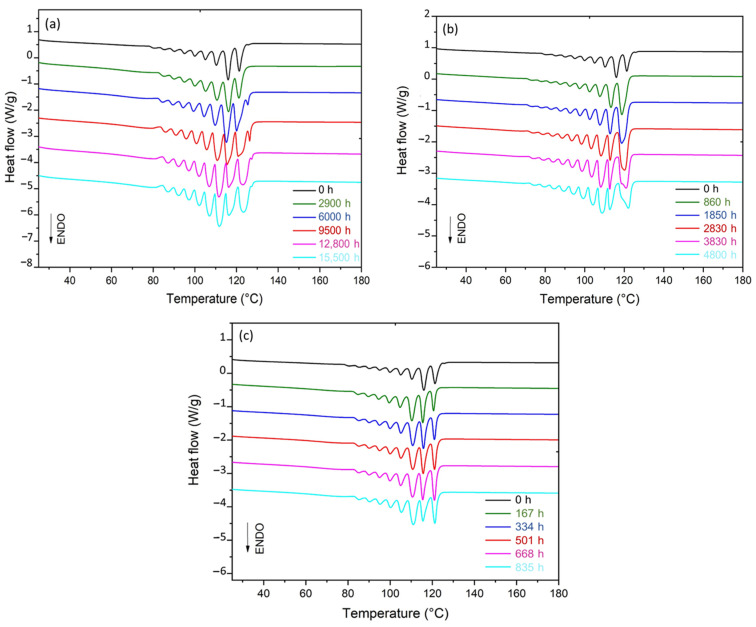
Fragmented DSC thermograms obtained after running the SSA method for Si-XLPE before and after its radiothermal ageing in air under (**a**) 8.5 Gy·h^−1^ at 47 °C, (**b**) 77.8 Gy·h^−1^ at 47 °C, and (**c**) 400 Gy·h^−1^ at 21 °C.

DSC was used with the SSA method to reveal the thickness distribution of crystalline lamellae in the Si-XLPE films and deduce an average value of lamellar thickness L_C_. Of course, L_C_ could directly be determined from the melting temperature T_m_ of the main endothermic peak on the DSC thermogram. However, as this melting peak was very broad, typically ranging from 25 °C to 125 °C, with two shoulders at about 40 °C and 80 °C, the peak maximum temperature may not obviously represent an average value close to the material microstructure. By running the SSA method, the melting domain was thermally split into a series of elementary melting peaks, with each one characterised by a distinct melting temperature, which can give a much better representation of the material microstructure. Figure 8 shows the fragmented DSC thermograms after applying the SSA method on the Si-XLPE films radiothermally aged in the air under 8.5 Gy·h^−1^ at 47 °C, 77.8 Gy·h^−1^ at 47 °C, and 400 Gy·h^−1^ at 21 °C.

**Figure 9:**
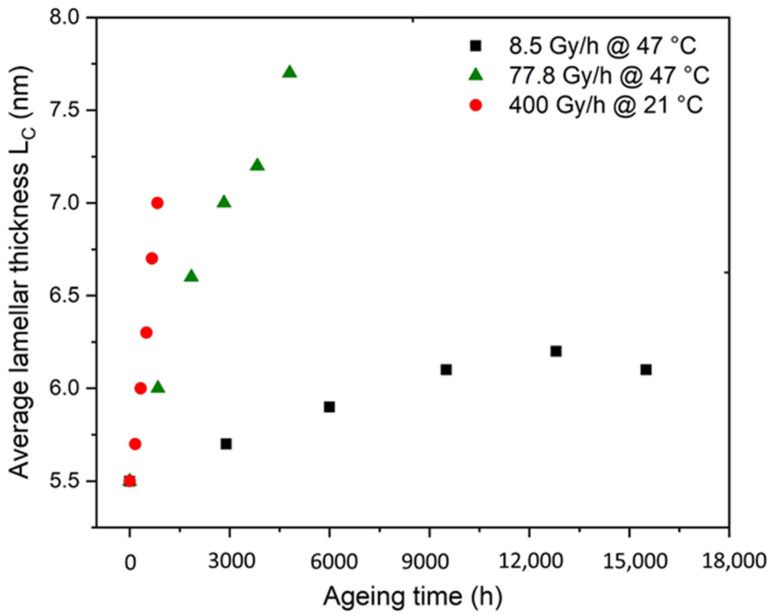
Changes in the average lamellar thickness determined via DSC for Si-XLPE during its radiothermal ageing in air under 8.5 Gy·h^−1^ at 47 °C (in black), 77.8 Gy·h^−1^ at 47 °C (in green), and 400 Gy·h^−1^ at 21 °C (in red).

The corresponding values of lamellar thickness L_Ci_ were calculated with the common Gibbs–Thomson’s equation (Equation (2)). Then, the average lamellar thickness L_C_ was deduced by using Equation (3). As shown in Figure 9, the changes in L_C_ were plotted as a function of the time of exposure in the air under 8.5 Gy·h^−1^ at 47 °C, 77.8 Gy·h^−1^ at 47 °C, and 400 Gy·h^−1^ at 21 °C. It was observed that L_C_ increased with the time of exposure for the three radiothermal exposure conditions under study. While for the lower dose rate (i.e., 8.5 Gy·h^−1^), L_C_ seemed to increase until reaching a plateau; in contrast, for the two other dose rates (i.e., 77.8 and 400 Gy·h^−1^), the exposure time seemed too short to achieve such a behaviour, even if higher values of L_C_ were measured. Nonetheless, these experimental data were closely related to the changes in the crystallinity ratio reported in Figure 7. A similar trend was indeed observed with increasing the time of exposure, regardless of the dose rate. As a consequence, the increase in crystallinity ratio can reasonably be attributed to the increase in lamellar thickness due to chemicrystallisation.

**Figure 10:**
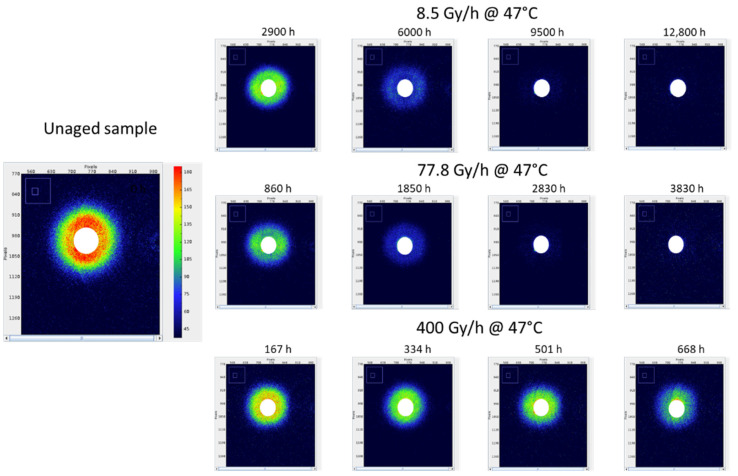
X-ray scattering patterns determined by SAXS for Si-XLPE during its radiothermal ageing in air under 8.5 Gy·h^−1^ at 47 °C (**top**), 77.8 Gy·h^−1^ at 47 °C (**middle**), and 400 Gy·h^−1^ at 21 °C (**bottom**). The white area in the centre corresponds to the mask used for covering the beam-stop area for the integration of the intensity versus the scattering vector.

SAXS measurements were performed to determine the characteristic dimensions of the polymer microstructure, namely the long period L_P_. Figure 10 reports the X-ray scattering patterns for the Si-XLPE films radiothermally aged in the air under 8.5 Gy·h^−1^ at 47 °C, 77.8 Gy·h^−1^ at 47 °C, and 400 Gy·h^−1^ at 21 °C.

**Figure 11:**
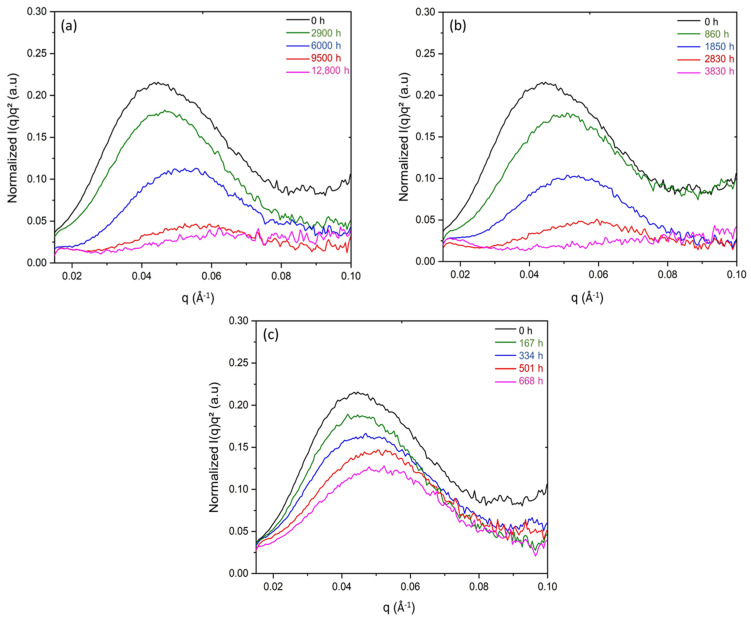
SAXS pattern of Si-XLPE before and after its radiothermal ageing in air under (**a**) 8.5 Gy·h^−1^ at 47 °C, (**b**) 77.8 Gy·h^−1^ at 47 °C, and (**c**) 400 Gy·h^−1^ at 21 °C.

It was clearly observed that the intensity of the SAXS pattern decreased progressively with the time of exposure until completely extinguishing after 12,800 h of exposure under 8.5 Gy·h^−1^ at 47 °C, and 3080 h under 77.8 Gy·h^−1^ at 47 °C. It is noteworthy that, for the highest dose rate (i.e., 400 Gy·h^−1^), the exposure time was too short to reach such a degradation state, as a correlation peak was still detected for the longest duration. As shown in Figure 11, the normalised intensity was then plotted as a function of the scattering vector q in order to quantitatively compare the different radiothermal ageing states.

**Figure 12:**
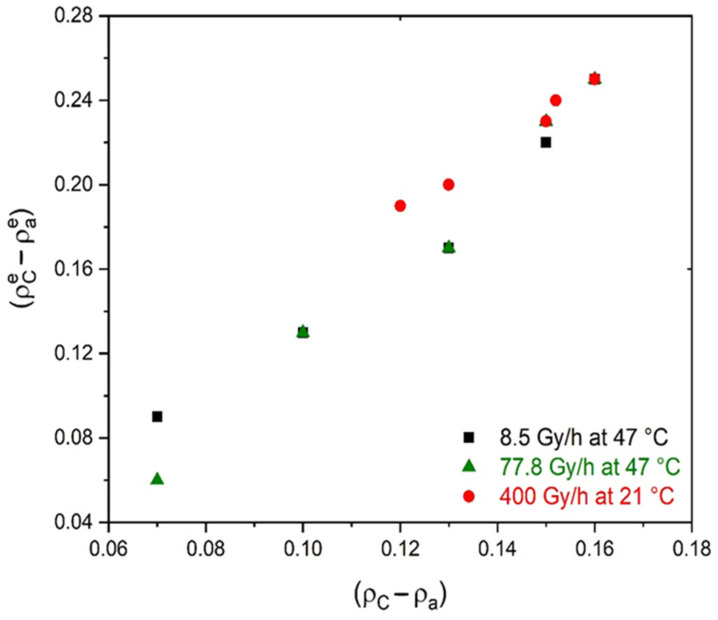
Changes in $\left(\rho_{c}^{e}-\rho_{a}^{e}\right)$ measured through SAXS as a function of $\left(\rho_{C}-\rho_{a}\right)$, measured through hydrostatic weighting for Si-XLPE radiothermally aged in air under 8.5 Gy·h^−1^ at 47 °C (in black), 77.8 Gy·h^−1^ at 47 °C (in green), and 400 Gy·h^−1^ at 21 °C (in red).

Before ageing, the SAXS pattern of the Si-XLPE film showed a broad correlation peak, corresponding to a long period of L_P_ = 14.2 nm. During ageing, it was observed that the γ irradiation caused both a decrease in the corrected intensity and a shift to higher values of the scattering vector position taken at the maximum of the correlation peak (q_max_), which indicated a decrease in L_P_. As can be observed in the SAXS patterns, the normalised intensity decreased with the time of exposure. In order to interpret this result, the difference in the electronic density between the amorphous and crystalline phases $\left(\rho_{c}^{e}-\rho_{a}^{e}\right)$ was plotted as a function of the difference in their density $\left(\rho_{C}-\rho_{a}\right)$. Figure 12 clearly shows a linear correlation between both quantities due to their simultaneous decrease with the time of exposure. The straight line intercepted the *y*-axis at a non-null value, probably due to the fact the intensity used in the SAXS experiment was too low to highlight an electronic contrast when the density of the amorphous phase approached that of the crystalline phase. The decrease in $\left(\rho_{C}-\rho_{a}\right)$ can be explained by the densification of the amorphous phase during radiothermal exposure. Indeed, as the crystalline phase is impermeable to oxygen, its density remains unaffected. Thus, oxidation reactions occur only in the amorphous phase, and oxygen consumption leads to a significant increase in its density, as shown in previous publications [9,10]. This important result explains the decrease in the intensity of the scattering pattern of Si-XLPE for the three radiothermal exposure conditions under study.

**Figure 13:**
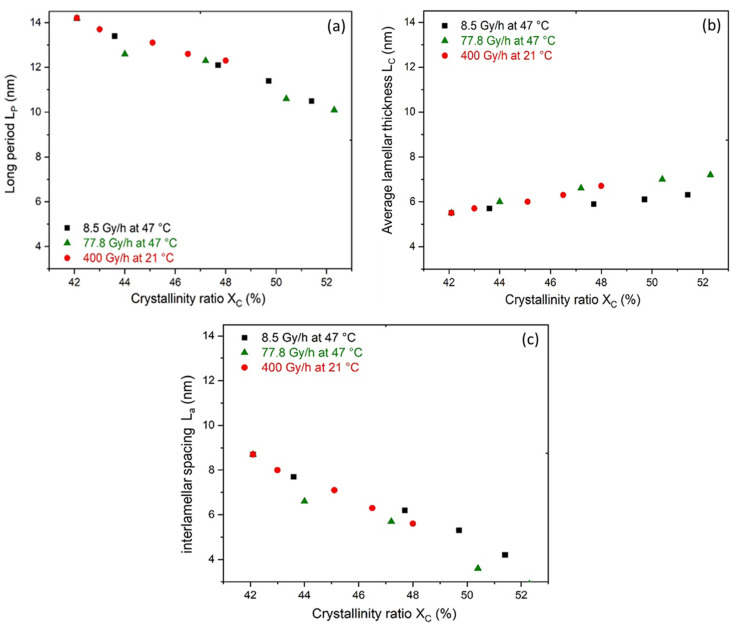
Changes in the (**a**) long period L_P_, (**b**) average lamellar thickness L_C_, and (**c**) interlamellar spacing L_a_ versus crystallinity ratio during the radiothermal ageing of Si-XLPE in air under 8.5 Gy·h^−1^ at 47 °C (in black), 77.8 Gy·h^−1^ at 47 °C (in green), and 400 Gy·h^−1^ at 21 °C (in red).

Knowing the average lamellar thickness L_C_ obtained through DSC according to the SSA method (Figure 9), the interlamellar spacing L_a_ was calculated using Equation (5). The values of L_a_ were plotted as a function of the crystallinity ratio X_C_ and are shown in Figure 13c.

It is noteworthy that L_P_ and L_a_ were decreasing functions, whereas L_C_ was an increasing function of X_C_. These changes had several consequences on the crystalline morphology and the mechanical behaviour of the Si-XLPE films for the three radiothermal exposure conditions under study. First of all, chemicrystallisation phenomena led to lamellar thickening and an increase in the crystallinity ratio, which caused the stiffening of Si-XLPE, as shown by micro-indentation experiments. In addition, the simultaneous decreases in L_P_ and L_a_ resulted in the confinement of the amorphous domains, thus leading to a loss in molecular mobility (i.e., the transition from a rubbery to glassy state) and necessarily having repercussions on the fracture properties of the Si-XLPE films (i.e., a change from ductile to brittle behaviour).

**Figure 14:**
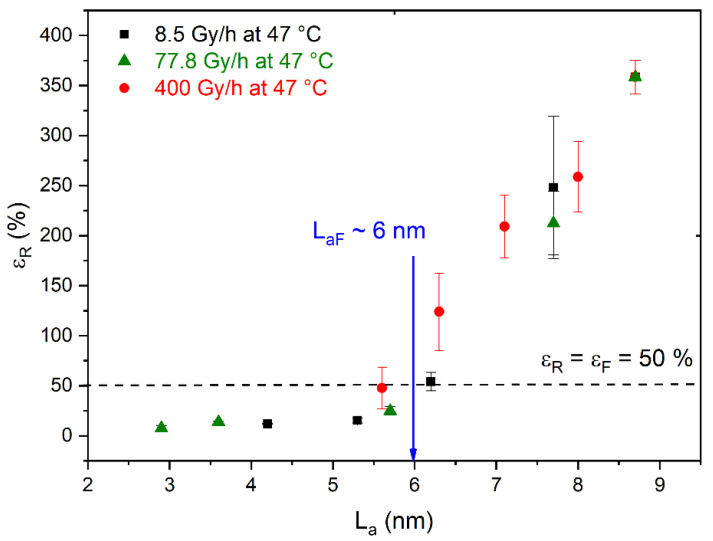
Elongation at break ε_R_ as a function of interlamellar spacing L_a_ for Si-XLPE radiothermally aged in air under 8.5 Gy·h^−1^ at 47 °C (in black), 77.8 Gy·h^−1^ at 47 °C (in green), and 400 Gy·h^−1^ at 21 °C (in red).

For this reason, the elongation at break ε_R_ of the Si-XLPE films, reported for the same radiothermal ageing conditions in previous publications [6,8], was plotted as a function of L_a_, as shown in Figure 14. It should be noted that these values of ε_R_ were determined with uniaxial tensile testing on H2-shaped dumb-bell specimens with a constant crosshead speed of 50 mm·min^−1^ at 23 °C under 50% RH. In Figure 14, it can clearly be observed that the couple (ε_R_, L_a_) was totally independent of the radiothermal exposure conditions. The corresponding master curve was then used for the graphic determination of a universally valid structural end-of-life criterion L_aF_ for Si-XLPE, closely related to the conventional end-of-life criterion for electrical cable insulation in the nuclear industry, i.e., t = t_F_ when ε_R_ = ε_F_ = 50%. A critical value L_a_ = 6 nm was thus determined for Si-XLPE. In fact, this value of L_aF_ happens to be of the same order of magnitude as that for linear PE, for which L_aF_ = 6–7 nm [20,21].

## 4. Conclusions

In this study, we investigated the effects of radiothermal ageing of additive-free silane-crosslinked polyethylene (Si-XLPE) at the crystalline morphological scale in order to propose a structural end-of-life criterion for lifetime prediction. DSC, WAXS, SAXS, and density measurements were performed to highlight the oxidation effects on the crystalline morphology of Si-XLPE. Both DSC and WAXS analyses revealed an increase in the crystallinity ratio X_C_ due to chemicrystallisation phenomena induced by chain scissions. It was found that the results obtained with these two techniques perfectly correlated. The increase in X_C_ was found to be accompanied by lamellar thickening, which was highlighted by using DSC together with the SSA method. In addition, SAXS measurements allowed determining the changes in the long period L_P_ from which the changes in the interlamellar spacing L_a_ were deduced (knowing the changes in the average lamellar thickness L_C_ through DSC). A decrease in both L_P_ and L_a_ was observed during radiothermal ageing, suggesting that the increase in L_C_ led to the confinement of the amorphous phase. Finally, the comparison between the results obtained with SAXS and uniaxial tensile testing allowed the determination of a critical value of interlamellar spacing as a structural end-of-life criterion for Si-XLPE: L_aF_ ≈ 6 nm. This critical value is of the same order of magnitude as that for linear PE. It can now be introduced into the kinetic models [6,8] recently developed for predicting the lifetime of Si-XLPE in a nuclear environment.

## Figures and Tables

**Table 1:** Physicochemical and mechanical properties of the as-received free-additive Si-XLPE films.

**Density** ρ	0.914 ± 0.002
**Melting temperature T_m_ ( °C)**	114.3 ± 0.4
**Crystallinity ratio X_C_ (%)**	42.1 ± 1.0
**Young’s modulus (MPa)**	260 ± 15
**Elongation at break (%)**	358 ± 17

**Table 2:** Radiothermal ageing conditions under study.

Dose Rate (Gy·h^−1^)	Dose Rate (Gy·s^−1^)	Temperature (°C)	Withdrawal Times of Samples (h)	Withdrawal Doses of Samples (kGy)
8.5	2.36 × 10^−3^	47	2900–6000–9500–12,800–15,500	25–51–81–109–132
77.8	2.16 × 10^−2^	47	860–1850–2830–3830–4800	67–144–220–298–373
400	1.11 × 10^−1^	21	167–334–501–668–835	68–134–200–267–334

## Data Availability

The data presented in this study are available on request from the corresponding author.
